# Impact of physical activity, health literacy and cognitive health on quality of life in dyslipidemia: an observational study

**DOI:** 10.1038/s41598-025-02223-4

**Published:** 2025-10-13

**Authors:** Furkan OZDEMIR, Melda SAGLAM, Oğuz A. UYAROGLU, Naciye VARDAR YAGLI

**Affiliations:** 1 Faculty of Health Sciences, Department of Physiotherapy and Rehabilitation, Çankırı Karatekin University, Çankırı, Türkiye; 2 Faculty of Physical Therapy and Rehabilitation, Department of Cardiopulmonary Physiotherapy and Rehabilitation, Hacettepe University, Ankara, Türkiye; 3 Faculty of Medicine, Department of Internal Disease, Hacettepe University, Ankara, Türkiye

**Keywords:** Cognitive health, Dyslipidemia, Health literacy, Physical activity, Quality of life, Medical research, Outcomes research

## Abstract

While statin therapy is primary intervention, lifestyle changes are also essential for managing blood lipids. Treatment success may be influenced by personal factors, such as health literacy, cognitive health, physical activity (PA). This study aimed to investigate the impact of PA, health literacy, and cognitive health over quality of life in dyslipidemia. Fifty-five patients were included in this study. Quality of life (36-Item Short Form Health Survey [SF-36]), health literacy (European Health Literacy Survey Questionnaire [HLS-EU-Q47]), cognitive status (Mini Mental State Examination [MMSE]), and PA (International Physical Activity Questionnaire–Short Form [IPAQ]) were evaluated. Physical functioning, physical role limitation, pain, general health, vitality, social functioning, emotional well-being sub-domains of SF-36 were associated with HLS-EU-Q47 (*p* < 0.05). Physical functioning, physical role limitation, pain and social functioning sub-domains of SF-36 were associated with MMSE (*p* < 0.05). Pain and social functioning sub-domains of SF-36 were associated with moderate PA score and daily sitting time of IPAQ, physical role limitation and emotional role limitation sub-domains of SF-36 were associated with walking score of IPAQ, emotional role limitation sub-domain of SF-36 was associated with total PA score of IPAQ (*p* < 0.05). Quality of life is an important indicator of general health, and enhanced health literacy and cognitive functions are associated with better quality of life in dyslipidemia. On the other hand, PA may be associated with quality of life, cognitive functions and health literacy in dyslipidemia. Increased health literacy and cognitive functions provide a better quality of life in dyslipidemia.

*Trial registration*: “Are Exercise Capacity and Physical Fitness Related With Quality of Life, Physical Activity, Cognitive Health and Health Literacy in Dyslipidemia” NCT06563154 (submitted at 17.08.2023 to clinicaltrials.gov).

## Background

Dyslipidemia is an important risk factor for cardiovascular diseases, atherosclerosis and cerebrovascular diseases^1,2^ Lifestyle change interventions are mostly effective for managing blood lipids. Lipid profile disorders are a major public health concern globally, affecting approximately one in three individuals^3,4^ Currently, ischemic heart diseases and central nervous system disorders are reported as the leading causes of mortality and morbidity in the adult population worldwide^4^.

The positive effects of PA on general health are well known and it is recognized as an effective approach to preventing mortality and improving quality of life^5,6^ PA levels are associated with various health conditions such as dyslipidemia and obesity^7^ Different studies showed that PA can decrease total cholesterol (TC), low density lipoprotein (LDL-C), triglycerides (TG), and increase high density lipoprotein (HDL-C)^1,2^.

Cognitive dysfunction, a major health problem which decreasing PA and quality of life in adult population worldwide^8^ Dyslipidemia, a risk factor for cerebrovascular diseases, can lead to Alzheimer’s disease and dementia^9^ Studies have shown a relationship between high TC, LDL-C and TG levels and cognitive impairment, especially increasing the risk of developing Alzheimer’s disease^10,11^ Prevention of cognitive dysfunction is one of the main goals for healthy aging worldwide^11,12^ Maintaining normal blood lipid levels and adopting lifestyle changes, including PA, are recommended to both preserve cognitive function and manage blood lipids^13^.

Health literacy describes as understanding, analyzing, and applying health-related information for different health problems and continuing healthy life^14^ Today, health literacy is accepted as a key component for both preventing chronic diseases and adherence to treatment. Studies showed that low health literacy is associated with increased morbidity and mortality. In addition, low health literacy is associated with unhealthy lifestyle behaviors such as being less physically active, poor nutritional habits, smoking, and being overweight, especially in patients with cardiovascular diseases^15,16^ Low health literacy is common in patients with cardiovascular diseases, especially patients with coronary artery diseases. Thus, enhancing health literacy is suggested as a main objective for improving patients’ health and preventing cardiovascular diseases^1,17^.

Despite the well-known relationship between PA and blood lipid levels, there are limited resources about the relationship between PA, cognitive functions, health literacy and quality of life in patients with dyslipidemia. Our aim was to investigate the relationship between physical activity, cognitive functions, health literacy and quality of life in patients with dyslipidemia.

This study hypothesized that health literacy, physical activity and cognitive health have an impact on quality of life in patients with dyslipidemia.

## Methods

### Study design

This study planned as cross-sectional design and was conducted in Hacettepe University Faculty of Physical Therapy and Rehabilitation between June and December 2023. The study was approved by the Local ethics committee on 03 January 2023 and with “03-01-2023-4” decision number. All participants were informed before the study and written informed consent was obtained from all participants. The study has also a clinical trial number (NCT06563154).

### Patients

Fifty-five patients were diagnosed with dyslipidemia but not receiving lipid management treatment (only lifestyle modifications) included in this study. The inclusion criteria for patients were being 18 to 65 years old, and volunteering for participation. The exclusion criteria for patients were having any cardiac, pulmonary, orthopedic, neurological, or psychiatric disease which may affect functional capacity or cooperation. Participants who were applied to hospital’s internal disease department for routine clinical examination were referred to center of research to including to the study, after informed consent. The study’s power was calculated post-hoc based on the correlation coefficient between the “General Health sub-parameter of SF-36” and “Total Score of HLS-EU-Q47” via G*Power (G*Power, ver. 3.1., Universität Düsseldorf, Düsseldorf, Germany) computer program. For 55 participants, 0.474 effect size and 5% type I error achieved power of the study was 96%.

### Measurements

All the measurements were conducted at the center of research via face-to-face interview. All measurements were assessed by same researcher for providing consistency. Cognitive health assessed firstly due to avoid cognitive fatigue, and health literacy, physical activity and quality of life assessed in this order.

#### Quality of life

Participants’ quality of life was evaluated with Turkish version of 36-Item Short Form Health Survey (SF-36)^18^ The SF-36 evaluates patient’s quality of life in 8 dimensions, which are limitation in activities because of physical health, limitation in activities because of emotional health, limitation in daily life roles because of physical health, limitation in daily life roles because of emotional health, perceiving pain, general mental health and emotional well-being, energy and fatigue perception, and general health perception. Higher scores shows better quality of life^19^.

#### Health literacy

Participants’ health literacy was evaluated with Turkish version of European Health Literacy Survey Questionnaire (HLS-EU-Q47)^20^ The HLS-EU-Q47 evaluates patients’ ability of decision making, reaching the information, understanding, and applying about three dimensions, which treatment, preventing from diseases, and improving health quality. All items’ scores as 1 to 4 points, and also has an option as “no idea”. The score of the questionnaire is calculated with the formula of “(mean score of all items – 1) * (50/3)”. Total score indicates which insufficient health literacy (0 to 25 point), limited health literacy (> 25 to 33 points), sufficient health literacy (> 33 to 42 points), and perfect health literacy (> 42 to 50 points). Higher HLS-EU-Q47 score shows better health literacy^20,21^.

#### Cognitive function

Participants’ cognitive function was evaluated with Turkish version of Mini Mental State Examination (MMSE)^22^ The MMSE evaluates patients’ understanding ability, adherence to the orders, time and location orientation, attention, calculating ability, and recording and remembering memory. Total score of MMSE calculating as total correct answer or doing correct order, and maximum score is 30 points. Higher MMSE score shows better cognitive capacity^22^.

#### Physical activity level

Participants’ PA level was evaluated with Turkish version of International Physical Activity Questionnaire – Short Form (IPAQ-SF)^23^ The IPAQ-SF evaluates PA according to vigorous PA, moderate PA, walking activity, and daily sitting time. IPAQ-SF consists of 7 items which assess the daily time of different severity activities, walking and sitting time at last 7 days. Total score calculates as sum of vigorous PA score, moderate PA score and walking score. High score means high PA level^23^.

### Statistical analysis

The appropriateness of the variables to normal distribution was analyzed by using Kolmogorov-Smirnov/Shapiro-Wilk tests-by visual (histogram and probability graphs) and analytical methods. Correlation between variables were evaluated using Pearson’s Correlation Test for normally distributed variables, and Spearman’s Correlation Test for non-normally distributed variables. Multiple linear regression model was performed using the sub-domains of SF-36 as the dependent variable and variables significantly related according to the correlation analysis (*p* < 0.005) as the independent variables, which were physical activity (IPAQ total score and daily sitting time), health literacy (HLS-EU-Q47 total score) and cognitive functions (MMSE score). Probability values of *p* < 0.05 were considered to be statistically significant.

## Results

Sixty-five patients with dyslipidemia were screened, and 10 patients were excluded because of rejecting participation. Demographic characteristics shown in Table 1. Participants’ mean SF-36 sub-domain scores are shown in Table 1. Participants’ mean physical function, role limitations due to physical problems, pain, and social function related quality of life were good (over 60 points), but general health, energy/vitality, role limitations due to emotional problems, and mental health related quality of life were poor (below 60 points) according to classification system of SF-36 questionnaire^24^ Participants’ mean vigorous and moderate intensity PA scores, walking score, and total score of IPAQ and mean daily sitting time values are shown in Table 1. Twenty-seven participants (49.1%) were in low PA category, 18 participants (32.7%) were in moderate PA category, and 10 participants (18.2%) were in high PA category according to IPAQ physical activity level classification^25^ Participants’ mean MMSE score is shown in Table 1. Nine participants’ (16.4%) MMSE score were below 24 points, which is cut-off point of MMSE in adults, and may have cognitive impairments^26^ Participants’ mean HLS-EU-Q47 score, and sub-domain scores are shown in Table 1. Six participants (10.9%) have insufficient health literacy, 23 participants (41.8%) have limited health literacy, 13 participants (23.6%) have sufficient health literacy, and 13 participants (23.6%) have perfect health literacy according to classification of HLS-EU-Q47^21^.

**Table 1:** Demographic characteristics, quality of life, physical activity, health literacy and cognitive functions of patients with dyslipidemia.

	Mean ± SD / Median (IQR) / *n* (%)
Age (years)	44.57 ± 11.94
Height (cm)	164.61 ± 9.16
Weight (kg)	76.71 ± 14.53
Body mass index (kg/m^2^)	28.49 ± 5.95
Smoking category	
Non-smoker	31(63.3%)
Ex-smoker	3(6.1%)
Active smoker	15(30.6%)
Smoke exposure (pack*years)	0 (7.5)
CRP (mg/dL)	4.32 ± 5.30
LDL-C (mg/dL)	140.73 ± 26.62
HDL-C (mg/dL)	53.27 ± 12.63
Total Cholesterol (mg/dL)	214.33 ± 32.75
Non-HDL Cholesterol (mg/dL)	177.32 ± 95.91
Triglycerides (mg/dL)	137.29 ± 57.91
Apolipoprotein-A1 (mg/dL)	45.34 ± 75.05
Fasting Blood Glucose (mg/dL)	93.57 ± 14.35
HbA1C (%)	5.90 ± 0.55
SF-36	
Physical Function (0-100)	79.82 ± 19.71
Role Limitation Due To Physical Health (0-100)	73.68 ± 36.72
Pain (0-100)	65.18 ± 26.90
General Health (0-100)	57.89 ± 20.74
Vitality/Energy (0-100)	54.91 ± 21.20
Social Functioning (0-100)	71.71 ± 25.17
Role Limitation Due To Emotional Problems (0-100)	57.90 ± 41.08
Emotional Well-being (0-100)	59.37 ± 19.72
HLS-EU-Q47	
Total Score (0–50)	34.89 ± 8.16
Health Care (0–50)	36.30 ± 8.04
Disease Prevention (0–50)	34.26 ± 8.83
Health Promotion (0–50)	33.50 ± 10.34
Mini Mental State Examination Score (0–30)	26.55 ± 3.50
IPAQ	
Vigorous Physical Activity Score (MET*min/week)	219.69 ± 795.30
Moderate Intensity Physical Activity Score (MET*min/week)	161.85 ± 419.74
Walking Score (MET*min/week)	670.92 ± 675.66
Total Physical Activity Score (MET*min/week)	1052.51 ± 1060.22
Daily Sitting Time (min/day)	270 ± 146.39

Mean ± SD: Mean ± Standard Deviation, IQR: Interquartile Renge, n: Total number, %: percentage, cm: centimeter, kg: kilogram, m: meter, dL: deciliter, MET: metabolic equivalent, CRP: C-Reactive Protein, LDL-C: Low Density Lipoprotein, HDL-C: High Density Lipoprotein.

Relationship between subdomains of SF-36 and IPAQ, HLS-EU-Q47 and MMSE are shown at Table 2. Physical functioning score of SF-36 showed positive correlation with total score (*r* = 0.459, *p* = 0.001), health care score (*r* = 0.491, *p* < 0.001), disease prevention score (*r* = 0.366, *p* = 0.007) and health promotion score (*r* = 0.437, *p* = 0.001) of HLS-EU-Q47, and MMSE score (*r* = 0.372, *p* = 0.006). Physical role limitation score of SF-36 showed positive correlation with walking score (*r* = 0.278, *p* = 0.036) of IPAQ and total score (*r* = 0.383, *p* = 0.005), health care score (*r* = 0.386, *p* = 0.004), disease prevention score (*r* = 0.331, *p* = 0.016) and health promotion score (*r* = 0.363, *p* = 0.008) of HLS-EU-Q47, and MMSE score (*r* = 0.398, *p* = 0.003). Pain score of SF-36 showed positive correlation with moderate PA score (*r* = 0.276, *p* = 0.037) and daily sitting time (*r* = 0.288, *p* = 0.030) of IPAQ, and total score (*r* = 0.452, *p* = 0.001), health care score (*r* = 0.470, *p* < 0.001), disease prevention score (*r* = 0.413, *p* = 0.002) and health promotion score (*r* = 0.394, *p* = 0.003) of HLS-EU-Q47, and MMSE score (*r* = 0.347, *p* = 0.01). General health score of SF-36 showed positive correlation with total score (*r* = 0.461, *p* = 0.001), health care score (*r* = 0.435, *p* = 0.001), disease prevention score (*r* = 0.387, *p* = 0.004) and health promotion score (*r* = 0.470, *p* < 0.001) of HLS-EU-Q47. Vitality score of SF-36 showed positive correlation with total score (*r* = 0.376, *p* = 0.006), health care score (*r* = 0.330, *p* = 0.016), disease prevention score (*r* = 0.369, *p* = 0.007) and health promotion score (*r* = 0.355, *p* = 0.009) of HLS-EU-Q47. Social functioning score of SF-36 showed positive correlation with moderate PA score (*r* = 0.275, *p* = 0.039) and daily sitting time (*r* = 0.296, *p* = 0.025) of IPAQ, and total score (*r* = 0.456, *p* = 0.001), health care score (*r* = 0.544, *p* < 0.001), disease prevention score (*r* = 0.428, *p* = 0.001) and health promotion score (*r* = 0.325, *p* = 0.017) of HLS-EU-Q47, and MMSE score (*r* = 0.525, *p* < 0.001). Emotional role limitation score of SF-36 showed positive correlation with walking score (*r* = 0.358, *p* = 0.006) and total PA score (*r* = 0.272, *p* = 0.041) of IPAQ. Emotional well-being score of SF-36 showed positive correlation with total score (*r* = 0.532, *p* < 0.001), health care score (*r* = 0.465, *p* < 0.001), disease prevention score (*r* = 0.525, *p* < 0.001) and health promotion score (*r* = 0.510, *p* < 0.001) of HLS-EU-Q47.

**Table 2:** Correlations between quality of life and health literacy, physical activity and cognitive health in patients with dyslipidemia.

			SF-36							
			Physical Functioning	Physical Role Limitation	Pain	General Health	Vitality/Energy	Social Functioning	Emotional Role Limitation	Emotional Well-being
IPAQ	Vigorous PA Score	r: p:	0.111 0.411	0.122 0.367	-0.023 0.867	0.048 0.722	-0.119 0.379	0.025 0.855	0.077 0.570	-0.197 0.142
	Moderate PA Score	r: p:	0.045 0.740	-0.061 0.654	0.276* **0.037**	-0.102 0.449	0.076 0.575	0.275* **0.039**	-0.050 0.713	-0.101 0.456
	Walking Score	r: p:	0.027 0.844	0.278* **0.036**	0.062 0.645	-0.133 0.323	-0.068 0.615	0.038 0.780	0.358* **0.006**	-0.022 0.869
	Total PA Score	r: p:	0.120 0.374	0.250 0.061	0.135 0.316	-0.092 0.497	-0.104 0.442	0.155 0.251	0.272* **0.041**	-0.205 0.126
	Daily Sitting Time	r: p:	-0.089 0.512	-0.224 0.095	-0.288* **0.030**	-0.035 0.798	-0.048 0.724	-0.296* **0.025**	-0.114 0.397	-0.111 0.412
HLS-EU-Q47	Total Score	r: p:	0.459* **0.001**	0.383* **0.005**	0.452* **0.001**	0.461* **0.001**	0.376* **0.006**	0.456* **0.001**	0.055 0.697	0.532* **< 0.001**
	Health Care	r: p:	0.491* **< 0.001**	0.386* **0.004**	0.470* **< 0.001**	0.435* **0.001**	0.330* **0.016**	0.544* **< 0.001**	0.094 0.503	0.465* **< 0.001**
	Disease Prevention	r: p:	0.366* **0.007**	0.331* **0.016**	0.413* **0.002**	0.387* **0.004**	0.369* **0.007**	0.428* **0.001**	0.026 0.852	0.525* **< 0.001**
	Health Promotion	r: p:	0.437* **0.001**	0.363* **0.008**	0.394* **0.003**	0.470* **< 0.001**	0.355* **0.009**	0.325* **0.017**	0.035 0.805	0.510* **< 0.001**
MMSE Score		r: p:	0.372* **0.006**	0.398* **0.003**	0.347* **0.010**	0.125 0.367	0.081 0.560	0.525* **< 0.001**	0.174 0.208	0.206 0.135

SF-36: 36-Item Short Form Health Survey, IPAQ: International Physical Activity Questionnaire, PA: Physical activity, HLS-EU-Q47: European Health Literacy Survey Questionnaire, MMSE: Mini Mental State Examination, r: Correlation Coefficient, *p < 0.05.

On the other hand, daily sitting time of IPAQ showed negative correlation with healthcare score of HLS-EU-Q47 (*r*=-0.274, *p* = 0.033) and MMSE (*r*=-0.304, *p* = 0.016). MMSE score showed positive correlation with total score (*r* = 0.453, *p* < 0.001), healthcare score (*r* = 0.535, *p* < 0.001), disease prevention score (*r* = 0.345, *p* = 0.006) and health promotion score (*r* = 0.378, *p* = 0.003) of HLS-EU-Q47.

Multiple linear regression models of the sub-domains of quality of life in patients with dyslipidemia are shown in Table 3. Health literacy independently predicted the physical function score of SF-36 (R^2^ = 0.254, F = 4.088, *p* = 0.006). Health literacy and cognitive health independently predicted the physical role limitation score of SF-36 (R^2^ = 0.289, F = 4.880, *p* = 0.002). Health literacy and daily sitting time independently predicted the pain score of SF-36 (R^2^ = 0.260, F = 4.212, *p* = 0.005). Health literacy independently predicted the general health score of SF-36 (R^2^ = 0.237, F = 3.725, *p* = 0.01). Health literacy, cognitive health and daily sitting time independently predicted the social function score of SF-36 (R^2^ = 0.355, F = 6.612, *p* < 0.001). Health literacy independently predicted the emotional well-being score of SF-36 (R^2^ = 0.306, F = 5.303, *p* = 0.001).

**Table 3:** Multiple linear regression model of the sub-domains of SF-36 in patients with dyslipidemia.

		*R*	*R* ^2^	*p*	B	β
Physical Function	IPAQ Total Score	0.120	0.014	0.374	0.002	0.120
	IPAQ Daily Sitting Time	0.089	0.008	0.512	0.013	-0.089
	HLS-EU-Q47 Total Score	0.447	0.200	0.003	1.188	0.447
	MMSE Score	0.291	0.084	0.056	1.557	0.291
	R^2^ = 0.254, F = 4.088, *p* = 0.006, Constant = 23.470					
Physical Role Limitation	IPAQ Total Score	0.250	0.062	0.061	0.009	0.250
	IPAQ Daily Sitting Time	0.224	0.050	0.095	0.061	-0.224
	HLS-EU-Q47 Total Score	0.372	0.139	0.014	1.670	0.372
	MMSE Score	0.337	0.114	0.025	3.118	0.337
	R^2^ = 0.289, F = 4.880, *p* = 0.002, Constant=-22.739					
Pain	IPAQ Total Score	0.135	0.018	0.316	0.003	0.135
	IPAQ Daily Sitting Time	0.288	0.083	0.030	0.058	-0.288
	HLS-EU-Q47 Total Score	0.424	0.180	0.005	1.415	0.424
	MMSE Score	0.272	0.074	0.074	1.870	0.272
	R^2^ = 0.260, F = 4.212, *p* = 0.005, Constant=-11.733					
General Health	IPAQ Total Score	0.092	0.008	0.497	-0.002	-0.092
	IPAQ Daily Sitting Time	0.035	0.001	0.798	0.005	-0.035
	HLS-EU-Q47 Total Score	0.412	0.170	0.006	1.200	0.412
	MMSE Score	0.057	0.003	0.714	0.333	0.057
	R^2^ = 0.237, F = 3.725, *p* = 0.010, Constant = 30.119					
Vitality/ Energy	IPAQ Total Score	0.104	0.011	0.442	-0.002	-0.104
	IPAQ Daily Sitting Time	0.048	0.002	0.724	0.008	-0.048
	HLS-EU-Q47 Total Score	0.350	0.123	0.021	0.969	0.350
	MMSE Score	0.011	0.000	0.943	0.062	0.011
	R^2^ = 0.162, F = 2.314, *p* = 0.071, Constant = 34.771					
Social Functioning	IPAQ Total Score	0.155	0.024	0.251	0.004	0.155
	IPAQ Daily Sitting Time	0.296	0.088	0.025	0.056	-0.296
	HLS-EU-Q47 Total Score	0.447	0.199	0.003	1.419	0.447
	MMSE Score	0.478	0.229	0.001	3.116	0.478
	R^2^ = 0.355, F = 6.612, *p* < 0.001, Constant=-32.191					
Emotional Role Limitation	IPAQ Total Score	0.272	0.074	0.041	0.010	0.272
	IPAQ Daily Sitting Time	0.114	0.013	0.397	0.035	-0.114
	HLS-EU-Q47 Total Score	0.087	0.008	0.578	0.441	0.087
	MMSE Score	0.174	0.030	0.260	1.818	0.174
	R^2^ = 0.128, F = 1.766, *p* = 0.151, Constant = 22.422					
Emotional Well-being	IPAQ Total Score	0.205	0.042	0.126	-0.004	-0.205
	IPAQ Daily Sitting Time	0.111	0.012	0.412	0.016	-0.111
	HLS-EU-Q47 Total Score	0.484	0.216	0.001	1.256	0.354
	MMSE Score	0.149	0.022	0.334	0.785	0.149
	R^2^ = 0.306, F = 5.303, *p* = 0.001, Constant = 21.032					

IPAQ: International Physical Activity Questionnaire, PA: Physical activity, HLS-EU-Q47: European Health Literacy Survey Questionnaire, MMSE: Mini Mental State Examination.

## Discussion

The most important finding of the present study is that health literacy, cognitive health and physical activity have an impact on different dimensions of quality of life, and well quality of life is associated with higher health literacy and cognitive health in patients with dyslipidemia.

Health literacy is an important factor for constructing and maintaining a healthy lifestyle. So, it is thought that well health literacy leads to the acquisition of healthy living habits that will improve the quality of life. Previous studies reported that higher health literacy is associated with better quality of life^27–29^ Our results align with these findings, demonstrating a correlation between higher health literacy and improved quality of life. All dimensions of quality of life, except role limitations due to emotional problems, were increased in patients with higher health literacy. Additionally, our results showed that health literacy may be a determining factor for physical functioning, physical role limitation, general health, pain perception, social functioning and emotional well-being dimensions of quality of life. This may be attributed to the fact that individuals with higher health literacy are more likely to adopt healthy behaviors related to harmful addictions (like smoking), PA, sedentary lifestyle, nutritional habits, and overall lifestyle, which can lead to improved quality of life in various aspects. On the other hand, higher heath literacy is strongly associated with better cognitive functions. This is not surprising, as individuals with higher health literacy generally possess higher cognitive abilities^30^ Our results showed that patients who have better cognitive functions have higher health literacy, same as the previous studies^31,32^ This could be caused by health literacy assessments including different types of cognitive assessments like capability of understanding, reasoning ability, making reasonable decisions about healthy lifestyle. And better function at these dimensions may only be possible with better cognitive capability.

Furthermore, quality of life encompasses physical, psychological, and social well-being. Therefore, changes in quality of life can directly affect cognitive functions^33^ Our results confirm this strong association between quality of life and cognitive functions, particularly in areas such as physical function, physical role limitation, social function, and pain perception, consistent with previous studies^34,35^ On the other hand, our result also showed that cognitive functions are one of the determinants of quality of life in patients with dyslipidemia, especially in physical role limitation and social functioning areas. Cardiovascular disease risk increases because of the atherosclerotic process, and cerebrovascular atherosclerosis in small cerebral vessels can contribute to white matter damage and cognitive dysfunction. So, it is thought that increased cardiovascular risk related with dyslipidemia, type II diabetes and hypertension cause cognitive impairments because of the same vascular inflammatory process, and cardiovascular disease risk factors may also be an independent risk factor for cognitive dysfunctions and dementia^36,37^ Previous studies reported that PA improves cognitive functions and enhances mental health. Conversely, decreased PA may lead to the development of cognitive dysfunction^38^ However, our findings showed that vigorous PA, moderate PA or walking may not have a significant relationship between cognitive functions in patients with dyslipidemia.

Physical activity is one of the most effective interventions for enhancing health state and preventing various chronic diseases^39^ Physical activity participation accepted as an instrument for decreasing adiposity and cardiometabolic disease risk, and increasing muscular strength and endurance, aerobic fitness and quality of life^40^ Especially, PA may decrease the ratio of premature cardiac deaths, because of sedentary lifestyle, nearly 20 to 30%^41^ Increased PA may increase quality of life in different patient groups^40^ However, the direct relationship between increased PA and quality of life may be complex due to variations in PA patterns and perceptions of “quality of life” across different cultures. Cultural and sociodemographic factors can hinder the generalization of the relationship between PA and quality of life^42^ Our study findings indicate that increased PA is associated with improved social functioning, reduced pain perception, and decreased limitations caused by physical and emotional problems. These results support the general association between PA and quality of life^42^ Health literacy, like PA, is considered an important factor in health promotion. Increased health literacy empowers individuals to take responsibility for their healthy lifestyles, while limited health literacy can lead to unhealthy lifestyle habits^43–45^ Hollmann et al. reported that most of the patients with dyslipidemia state that the increased risk of cardiovascular diseases with hypercholesterolemia, few are aware of the actual effects of hypercholesterolemia, its management, their own blood lipid levels, or their cardiovascular disease risk^46^ According to general opinion, increased PA may be associated with increased health literacy^47^ However, our results suggest that PA and health literacy may not be correlated in patients with dyslipidemia. This discrepancy could be attributed to the fact that nearly 54% of our participants exhibited insufficient health literacy, and these individuals may not have a clear understanding of the positive effects of PA on their health. Although all participants reported receiving PA recommendations from their physicians or public service announcements, their health literacy and daily activity patterns were not assessed. Consequently, all participants received similar PA suggestions, potentially limiting the effectiveness of these recommendations. On the other hand, our results showed that daily sitting time has an impact on pain perception and social functioning sub-domains of quality of life.

As reported in numerous studies, PA is a protective factor against cardiovascular diseases due to its ability to lower blood lipids, particularly LDL-C, TG, and TC, and also it is thought that there is a link between increased blood lipids and decreased PA^1,2,48^ While cognitive functions are not expected to differ significantly in patients with dyslipidemia^49^, our study did not reveal any significant impairments in cognitive functions in this patient group. Lipid profile disorders are one of the most common causes of cardiovascular diseases. And also, low health literacy is associated with cardiovascular disease risk and increased mortality^50^ Health literacy is the capacity of individuals to understand medical information and directions, and applying the instructions of health professionals^14^ And it is thought that low health literacy may be associated with increased blood lipids by adapting unhealthy behavior^51^.

## Limitations

All assessments conducted by self-reported questionnaires in this study but more objective measurement instruments (like activity monitors for physical activity assessments) may provide more reliable data. The cross-sectional design of this study may not allow assessing the long-term alteration of the quality of life and the confounding factors. Thus, future research should assess the long-term changes in quality of life and the confounding factors of quality of life. The relationship between quality of life and physical activity, health literacy, cognitive health were highlighted in this study but the underlying mechanisms which may affect quality of life are not examined. So, future studies should explore the relationship between confounding factors and if affect quality of life subsequently in patients with dyslipidemia. The single-center design of the study may affect the generalizability of the findings. So, future studies should be designed as multi-center research study.

## Conclusions

Our study showed that increased quality of life is associated with higher health literacy, better cognitive functions and increased physical activity in patients with dyslipidemia. Additionally, nearly all dimensions of quality of life is a determining factor of health literacy, and social functioning has an impact on cognitive health, and physical role limitation has an impact on physical activity. On the other hand, health literacy has a relationship with cognitive health.

## Data Availability

The datasets used and/or analysed during the current study are available from the corresponding author on reasonable request.

## Abbreviations

ACG: Aerobic + calisthenic exercise group
AG: Aerobic exercise group
CG: Control group
dL: Deciliter
HDL-C: High-density lipoprotein
HLS-EU-Q47: European health literacy survey questionnaire
IPAQ-SF: International physical activity questionnaire – short form
LDL-C: Low-density lipoprotein
mg: Milligram
MMSE: Mini mental state examination
PA: Physical activity
SF-36: 36-Item short form health survey
TC: Total cholesterol
TG: Triglycerides
